# Genetic disorder or toxoplasma myocarditis: a case report of dilated cardiomyopathy with hypertrabeculation in a young asymptomatic woman

**Published:** 2012-03-05

**Authors:** M Dobranici, A Buzea, R Popescu, L Chirila

**Affiliations:** * Cardiology Department, Colentina University Hospital, Bucharest; ** ”Carol Davila” University of Medicine and Pharmacy, Bucharest

**Keywords:** cardiomyopathy, noncompaction, trabeculae, toxoplasmosis

## Abstract

Isolated noncompaction of the left ventricle (LV) is a rare disorder, classified as a primary genetic cardiomyopathy by the American Heart Association. The European Society of Cardiology Working Group on Myocardial and Pericardial Diseases classified LV noncompaction as an unclassified cardiomyopathy. LV noncompaction cardiomyopathy characterized by the following features: 1) an altered myocardial wall with prominent trabeculae and deep intertrabecular recesses resulting in thickened myocardium with two layers, consisting of compacted and noncompacted myocardium and 2) continuity between the left ventricular cavity and the deep intertrabecular recesses, which are filled with blood from the ventricular cavity, without evidence of communication with the epicardial coronary artery system. Features of LV noncompaction can overlap with dilated cardiomyopathy, hypertrophic cardiomyopathy (especially the apical variant), and restrictive cardiomyopathy. The phenotypic expression can vary considerably within the same family. The LV noncompaction can rarely occur as a transient phenomenon during myocarditis.

We present the case of a 23-year-old patient, admitted to our Department for cardiac evaluation because of ECG changes and cardiac enlargement revealed at thoracic radiography. She had a history of chronic toxoplasmosis. An echocardiography was performed revealing left ventricular enlargement with severe systolic and diastolic dysfunction, diffuse hypokinesia and signs of isolated left ventricular non-compaction. Under these circumstances, we have considered the presence of isolated left ventricular non-compaction. A cardiac Magnetic Resonance Imaging was performed and it sustained the diagnosis. The alternative cause of isolated left ventricular noncompaction (prominent trabeculation due to myocardial toxoplasmosis) was considered improbable.

## Introduction

The left ventricular (LV) isolated non-compaction is characterized by embryonic myocardium morphology due to an arrest in the compaction process during the first trimester, and it is a very rare cardiomyopathy. Clinical manifestations are non-specific, ranging from asymptomatic patients to acute congestive heart failure. The diagnosis is based on cardiac imaging using echocardiography and magnetic resonance imaging.

## Case report

A healthy 23-year-old woman presented to hospital for cardiac workup after she had been referred to a cardiologist by a general practitioner, after detecting changes on electrocardiogram (ECG), consisting of negative T waves and cardiac enlargement on thoracic radiography. She was asymptomatic at presentation and without any familial cardiac history. She had a history of chronic toxoplasmosis (with fever and lymphadenopathy, 12 months prior to this hospitalization) for which she was taking antibiotic treatment. The clinical examination showed pallor of skin and mucous membrane, maculo-papular non-itching rash on the forearms and thighs. There were also mobile axillary enlarged (2 cm) lymph nodes not tender or painful with moderate hard consistency, hepatosplenomegaly, blood pressure (BP) of 110/60 mmHg, heart rate (HR) of 70 b/min, regularly and apexian systolic murmur grade III/VI radiating to the axilla. Laboratory tests showed a discrete inflammatory syndrome (elevated erythrocyte sedimentation rate and C-reactive protein level), IgG antibodies to Toxoplasma in serum (1497,3 UI/mL). 

**Fig. 1:**
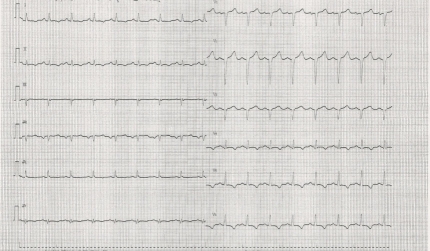
ECG at presentation

**Fig. 2:**
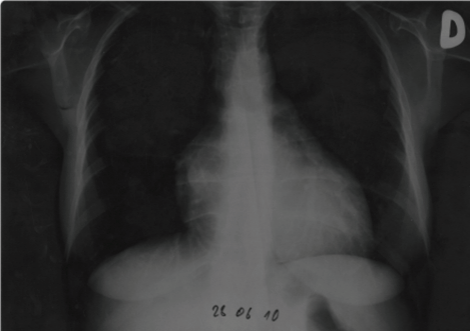
Chest radiography revealed heart enlargement

Initial ECG (see **Figure 1**) showed poor r wave progression in V1-V3 leads and negative T waves in DI, DII, aVF, and V4-V6 leads and thoracic radiography (see **Figure 2**) showed an increased cardio-thoracic index.

An echocardiogram (see **Figure 3 A, B, **and **C**) was performed revealing left ventricular enlargement with severe systolic and diastolic dysfunction (LV ejection fraction 25%, filling pattern type III- restrictive pattern), diffuse hypokinesia and trabeculation suggesting isolated left ventricular non-compaction.

**Fig. 3A:**
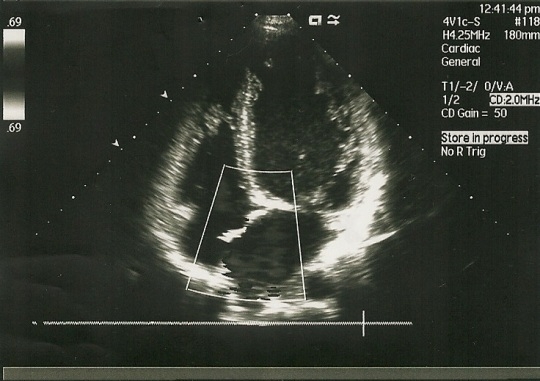
Echocardiography: LV trabeculation

**Fig. 3B:**
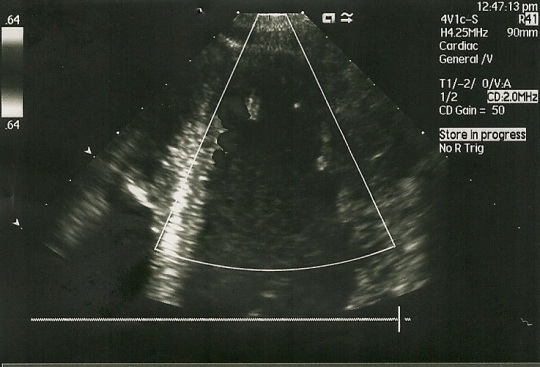
Echocardiography: LV magnified view of trabeculae

**Fig. 3C:**
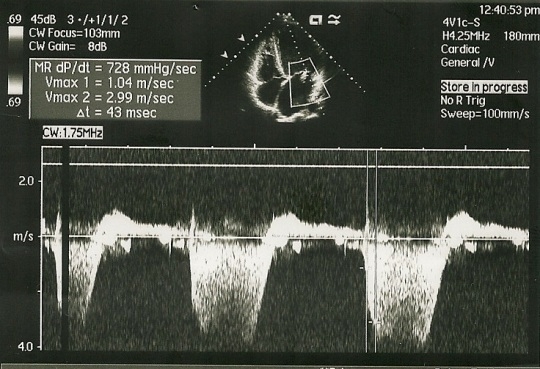
LV filling pattern at echocardiography

A cardiac Magnetic Resonance Imaging (MRI) (see **Figure 4 Aand  B**) was performed and it confirmed dilated cardiomyopathy with excessively prominent trabeculations in left lateral wall and ventricular apex, severe alteration of left ventricular function with diffuse hypokinesia.

**Fig. 4A:**
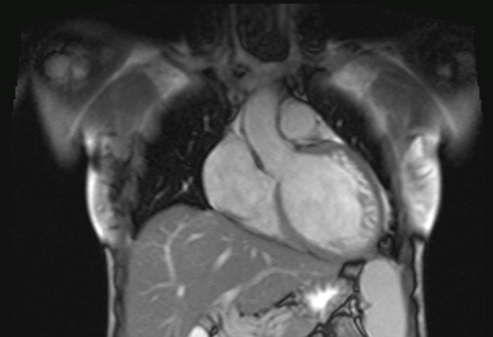
Visualization of trabeculae at MRI (frontal view)

**Fig. 4B:**
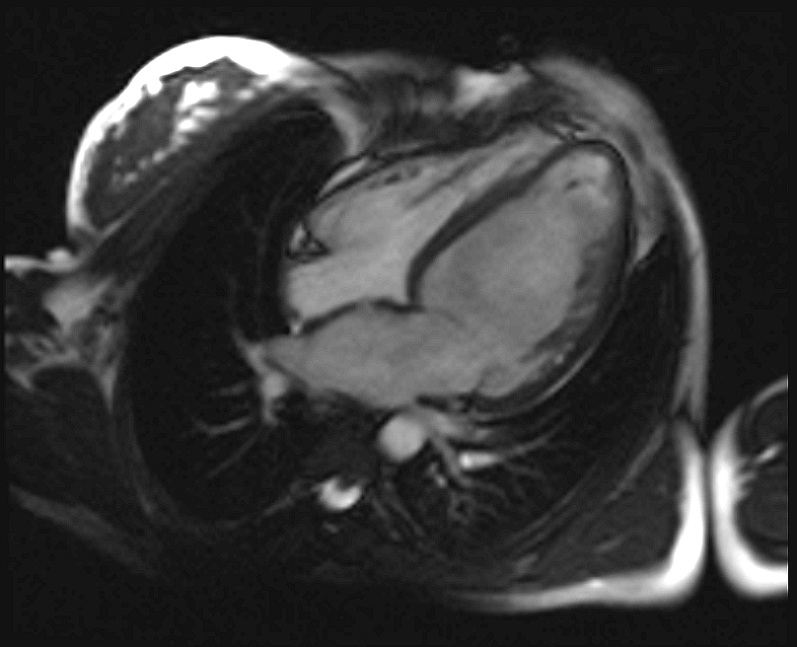
Visualization of trabeculae at MRI (sagital view)

## Discussion

One of the most important questions in this case is the etiology of dilated cardiomyopathy: a non-compacted or a secondary dilated cardiomyopathy. The left ventricular isolated non-compaction is a genetic cardiomyopathy and can occur isolated or in association with congenital heart defects, genetic syndromes and neuromuscular disorders. It is characterized by prominent trabeculations and deep recesses within the ventricular myocardium resulting in apparently hypertrophied myocardium. The myocardium is actually thickened and has two layers consisting of compacted and non-compacted myocardium [**1, 2, 3**]. Normal myocardium can present trabeculae but always less than three, while in non-compaction, the trabeculae are increased in number and very prominent, the compact layer is thinner and between the trabeculae there are deep recesses in communications with the ventricular cavity, but not with the epicardial coronary system [**3, 4, 5, 6**].

Most patients have systolic left ventricular dysfunction and they present with congestive heart failure. The diagnosis is given by the echocardiography. Current echocardiographic criteria include: a) presence of multiple trabeculae – especially in the apex and the left ventricular free wall; b) multiple deep recesses, between trabeculae, communicating with ventricular cavity (demonstrated by color Doppler imaging); c) a two-layer structure of the myocardium with non-compact increased layer (ratio between non-compact and compact layer suggested as >2.0) [**5, 6**]..

It is also known that left ventricular dilation, hypertrophy and systolic left ventricular dysfunction can present trabeculae and increased left ventricular bands. This is an apparent non-compaction and it can be difficult to differentiate by imagistic examination, even by using the proposed criteria [**3**]. A cause for dilated cardiomyopathy is known to be infectious myocarditis. The toxoplasma infection has a worldwide distribution and the primary infection is, in most cases, asymptomatic. The involvement of the myocardium is rare in immune-competent hosts and a development of secondary dilated cardiomyopathy has not yet been described. Several cases of toxoplasma myocarditis were published in the past. In 1954, Paulley et al [**7**], concluded that myocardial toxoplasmosis should be considered in all obscure cases of myocarditis and unexplained cardiac hypertrophy. In 1967, Arturo Arribada and Edgardo Escobar [**8**] concluded in a small study that cardiac failure might be due to a late complication of the toxoplasmic infection of heart and it is important that the proper diagnosis is made as early as possible. Despite these early reports we have not found any recent published cases of toxoplasma myocarditis in immune-competent host.

Despite her medical history of chronic toxoplasmosis, the diagnosis we consider for our patient was non-compaction cardiomyopathy. We based our diagnosis on: *absence of clinical manifestation* regarding any episode of acute myocarditis; *current clinical state *- asymptomatic patient suggesting long term development of cardiac enlargement; *cardiac imaging *–echocardiographic and MRI elements for non-compaction cardiomyopathy; *absence of suggestive literature data* regarding dilated cardiomyopathy secondary to toxoplasma infection in immune-competent host;* absence of regression*, under treatment, suggesting genetic disorder instead of apparent non-compaction.

An endomyocardial biopsy would have been useful, but presently, we have encountered technical difficulties along with the patient’s refusal and also genetic testing, both not being available yet.

As in previous reported cases [**9**], there is no specific treatment in noncompaction cardiomyopathy. We have been closely monitoring her condition with clinical examination, ECG and echocardiography and, so far, there have been no changes; currently she remains asymptomatic and she is treated with a beta blocker, an angiotensin-converting enzyme inhibitor, an oral anticoagulant and an implantable cardioverter-defibrillator were proposed for primary prevention of sudden cardiac death.
